# New‐onset Kleine–Levin syndrome following COVID‐19 infection: A case report

**DOI:** 10.1002/pcn5.70199

**Published:** 2025-08-29

**Authors:** Daisuke Yoshioka, Takehiko Yamanashi, Masaaki Iwata

**Affiliations:** ^1^ Division of Neuropsychiatry, Faculty of Medicine Tottori University Yonago Japan

**Keywords:** COVID‐19, hypersomnolence, Kleine–Levin syndrome, sleep disorder

## Abstract

**Background:**

Kleine–Levin syndrome (KLS) is a rare sleep disorder marked by recurrent episodes of severe hypersomnolence with accompanying cognitive, behavioral, or mood disturbances. Infections are frequently reported before symptom onset and have been proposed as potential triggers, although a definitive causal relationship has not been established. Although relapses of KLS after COVID‐19 infection have been described, only one possible case of new‐onset KLS associated with COVID‐19 has been reported, in which a definitive diagnosis was not established.

**Case Presentation:**

We describe a 17‐year‐old male who developed KLS following a confirmed COVID‐19 infection. After his clinical recovery, he began experiencing hypersomnolent episodes every few months, each lasting approximately 2 weeks and accompanied by decreased motivation and appetite. As the illness progressed, he developed persistent nausea lasting several weeks before each hypersomnolent episode, suggesting the emergence of a prodromal phase. Treatment with lithium and adjunctive modafinil showed limited efficacy in preventing recurrence or reducing episode severity. Each complete cycle comprising prodromal, hypersomnolent, and recovery phases lasted over a month and significantly disrupted his academic and social functioning.

**Conclusion:**

This case represents the first diagnostically confirmed report of new‐onset KLS following COVID‐19 infection and suggests a possible autoimmune mechanism triggered by SARS‐CoV‐2. These findings emphasize the importance of comprehensive clinical assessment beyond objective test results and underscore the urgent need for effective treatment strategies tailored to the heterogeneous and disabling nature of KLS.

## BACKGROUND

Kleine–Levin syndrome (KLS) is a rare sleep disorder marked by recurrent episodes of severe hypersomnia, often accompanied by a combination of cognitive, mood, and behavioral disturbances such as hyperphagia, hypersexuality, confusion, and emotional blunting.^1^, ^2^ Episodes typically last from a few days to several weeks, with intervening periods of normal sleep, cognition, and behavior.^3^ The condition primarily affects adolescent males and has an estimated prevalence of 1–5 cases per million individuals.^4^ The frequency, intensity, and progression of episodes vary between patients, contributing to the clinical complexity of the disorder.^5^

KLS was first described in 1925 by Willi Kleine, who reviewed nine previously reported cases and added five patients presenting with episodic somnolence.^6^ Nearly a century later, the pathophysiological mechanisms of KLS remain poorly understood. Despite accumulating case reports and clinical observations, no definitive biomarkers or structural abnormalities have been identified, and diagnosis continues to rely on clinical criteria and the exclusion of other etiologies.

Several pathophysiological mechanisms have been proposed for KLS, including a possible autoimmune origin. This hypothesis is supported by observations that hypersomnolent episodes are frequently preceded by mild infections, as well as by the relapsing–remitting nature of the disorder.^7^ More recently, several cases of KLS relapse and a suspected case of new‐onset KLS have been reported following SARS‐CoV‐2 (COVID‐19) infection,^8^, ^9^, ^10^ raising the possibility that this virus may act as a trigger for central hypersomnia. These findings are particularly relevant given the known effects of COVID‐19 on the central nervous system, including neuroinflammation and disruption of sleep–wake regulation.^11^ Such observations not only contribute to hypotheses regarding KLS pathophysiology but also underscore the need for clinical awareness of this rare condition.

Although KLS is rare, accurate diagnosis is essential, as it may be mistaken for psychiatric illness, encephalopathy, or other sleep disorders. Given that hypersomnolence is a symptom shared with several conditions, including narcolepsy, idiopathic hypersomnia, mood disorders, and neurologic diseases, careful evaluation of episode duration, associated symptoms, and inter‐episode recovery is vital for differential diagnosis. We report a case of new‐onset KLS following confirmed COVID‐19 infection in a 17‐year‐old male.

## CASE PRESENTATION

A 17‐year‐old male with no prior medical or psychiatric history and no significant family history contracted COVID‐19 in August, during the summer of his third year of high school. He initially presented with fever and sore throat, and a low‐grade fever persisted for about 1 week. After clinical recovery, he began to exhibit hypersomnolence, sleeping more than 12 h daily. Before infection, he had a regular sleep schedule, averaging 7 h per night.

Three months after symptom onset, he visited a sleep clinic for further evaluation. A multiple sleep latency test (MSLT) revealed a mean sleep latency of 5.5 min, with no sleep‐onset rapid eye movement (REM) periods observed during four nap opportunities (0/4 SOREMPs). Based on these findings, idiopathic hypersomnia was diagnosed, and modafinil was prescribed, but it provided little benefit. Pemoline and aripiprazole were added sequentially but were also ineffective. One year after onset, he was evaluated at another sleep clinic, where modafinil was continued, though hypersomnolence persisted. Due to overlapping hypersomnolence and low motivation, a mood disorder was suspected, and he was referred to psychiatry 16 months after onset. However, psychiatric evaluation ruled out depression and other psychiatric conditions, and his management returned to the sleep clinic.

During this period, he experienced hypersomnolent episodes every few months, each lasting about 2 weeks. During episodes, he slept 14 to over 20 h daily and showed marked reductions in motivation and appetite. Residual cognitive dysfunction, such as impaired attention and alertness, persisted for several weeks after each episode, compromising academic performance. Twenty‐one months after initial symptom onset, he experienced a recurrence and was referred to our hospital's Department of Psychiatry. At admission, his body weight had decreased from 55 kg to about 40 kg. He was admitted for evaluation and treatment. Blood tests, brain magnetic resonance imaging, and electroencephalography revealed no abnormalities. Based on the recurrent nature of hypersomnolent episodes, associated behavioral and physical symptoms, full symptom resolution between episodes, and exclusion of other medical, neurological, and psychiatric conditions, the patient was diagnosed with KLS. Testing for autoimmune‐related antibodies and cerebrospinal fluid analysis were not performed, as the clinical course was highly consistent with KLS, and previous studies have not identified disease‐specific autoantibodies or cerebrospinal fluid findings in this condition. Given the clear temporal link with prior COVID‐19 infection, the clinical course was considered consistent with KLS triggered by SARS‐CoV‐2. His hypersomnolence resolved spontaneously within 2 weeks, and he was discharged with plans for outpatient follow‐up. Lithium carbonate was started at 400 mg/day to prevent future episodes.

During the inter‐episode period, polysomnography (PSG) was performed. Total sleep time was 490 min; sleep latency was 4.9 min; and REM latency was 51.5 min. Sleep architecture was within normal limits, with appropriate proportions of non‐REM and REM stages. A repeat MSLT conducted the next day showed a mean sleep latency of 4.6 min and two SOREMPs in five naps. Although these PSG and MSLT results met the diagnostic criteria for narcolepsy, the diagnosis was not revised, as the episodic course, accompanying symptoms such as motivational decline and appetite loss, and absence of narcolepsy‐specific features were more consistent with KLS.

As episodes continued, lithium was titrated to 800 mg/day, and serum lithium reached 1.1 mEq/L. However, episodes still recurred every few months. Modafinil was added as adjunctive therapy and administered at the onset of each episode to alleviate symptoms, but no significant improvement was observed. In later episodes, the patient developed persistent nausea for several weeks before hypersomnolence, consistently preceding each episode and suggesting a recognizable prodromal phase.

Despite treatment, the patient continues to experience hypersomnolent episodes every few months. Each begins with several weeks of nausea, followed by about 2 weeks of hypersomnolence with motivational decline and appetite loss, leading to weight loss. This is followed by cognitive dysfunction marked by reduced attention and alertness, lasting several more weeks. Including prodromal and recovery phases, each illness cycle spans over a month, causing significant disruption to his daily life and academic functioning.

## DISCUSSION

This case illustrates a clinically meaningful presentation of new‐onset KLS following COVID‐19 infection. Although two reports have documented relapses of KLS triggered by COVID‐19,^8^, ^9^ confirmed cases of de novo onset remain extremely rare, with only a single unconfirmed report to date.^10^ Although these reports, including ours, share the common feature of KLS onset or relapse in temporal association with COVID‐19 infection, some have described abnormal findings such as white matter hyperintensities on fluid‐attenuated inversion recovery MRI or human herpesvirus 6–positive cerebrospinal fluid. However, none of these abnormalities are disease‐specific, and no consistent clinical characteristics have yet been identified in post–COVID‐19 KLS.

Infections are the most frequently reported triggers for both the onset and relapse of KLS. In a review of 475 published cases, precipitating factors were reported in 63.2%, with infections, mainly unspecified or upper airway types accounting for roughly 60%.^5^ These findings strongly support the clinical relevance of infections as the most common external trigger in KLS. Given the close temporal link between infection and symptom onset in our case, and the absence of prior neurological or psychiatric history, it is plausible that SARS‐CoV‐2 triggered KLS in a genetically or immunologically susceptible individual. This observation aligns with hypotheses suggesting postinfectious or autoimmune mechanisms in KLS pathophysiology,^7^, ^12^ in which infections may provoke immune responses, such as molecular mimicry or activation of autoreactive T cells that lead to symptom onset.^2^ Supporting this theory, cerebrospinal fluid and inflammatory markers, including C‐reactive protein, are typically normal during symptomatic episodes. Additionally, prior studies have shown no significant elevation of inflammatory cytokines during KLS episodes compared to inter‐episode phases.^12^, ^13^, ^14^ In line with these findings, blood tests in our case were also unremarkable. Taken together, these data make a direct infectious or inflammatory cause less likely and instead support immune dysregulation triggered by infection as a plausible mechanism. However, the effects of COVID‐19 on the central nervous system remain poorly understood, and a causal relationship in this case cannot be definitively established. In addition, persistent symptoms following COVID‐19 are collectively referred to as “long COVID,” a condition in which various symptoms, including fatigue, cognitive dysfunction, and sleep disturbances, continue beyond the acute phase of infection.^15^ Excessive daytime sleepiness has been reported in approximately 35.8% of patients with long COVID.^16^ However, sleepiness in long COVID typically does not involve the prolonged sleep durations seen in KLS. Moreover, long COVID generally follows a chronic, continuous course, whereas KLS is characterized by discrete, recurrent episodes with complete recovery between them. This episodic nature is a key clinical feature that helps distinguish KLS from long COVID.

In this case, MSLT revealed a mean sleep latency under 8 min and two or more SOREMPs, meeting the diagnostic criteria for narcolepsy based on the *Interna*t*ional Classification of Sleep Disorders, Third Edition – Text Revision* (*ICSD‐3‐TR*).^17^ Although SOREMPs are characteristic of narcolepsy, they have also been inconsistently observed in some KLS patients and are not considered diagnostic in this context.^18^

The patient experienced recurrent hypersomnolent episodes, each lasting several weeks, with full cognitive and behavioral recovery between episodes. Unlike narcolepsy, which typically presents as brief, daily sleep attacks lasting minutes to an hour, KLS involves prolonged hypersomnolence of 15–24 h per day, lasting for days to weeks.^12^ Although both disorders feature persistent daytime sleepiness, the prolonged, uninterrupted nature of hypersomnolence in KLS is distinct. Furthermore, symptoms commonly associated with narcolepsy, such as cataplexy or hypnopompic hallucinations, were absent, and no alternative medical, neurologic, psychiatric, or pharmacologic explanations were identified. Taken together, we diagnosed this patient with KLS.

This case also featured notable appetite disturbance. Although hyperphagia is classically linked to KLS, approximately one‐third of patients instead exhibit reduced appetite during episodes, and such cases have been reported to present with more severe hypersomnolence than those with hyperphagia.^19^ In our case, the patient exhibited marked anorexia and near‐complete cessation of food intake during symptomatic periods, resulting in significant weight loss. This pattern may suggest a worse prognosis in terms of severity and chronicity. Additionally, the patient developed episodes of persistent nausea during the course of illness, each occurring shortly before the onset of hypersomnolent episodes. Since both lithium and modafinil had been in use for an extended period without prior adverse effects, a medication‐related cause of the nausea was considered unlikely. Given its consistent temporal association with episode onset, the nausea was regarded as a possible prodromal feature of KLS, although this has not been consistently emphasized in the literature. In considering differential diagnoses, cyclothymia is one condition that may share certain overlapping features with KLS. Cyclothymia is a chronic mood disorder characterized by recurrent mild hypomanic and depressive symptoms over a prolonged period, sometimes accompanied by changes in sleep and appetite.^20^ Although KLS and cyclothymia share several features, including a recurrent and periodic course, sleep disturbances, mood changes, alterations in appetite, and the consideration of lithium as a treatment option, their primary manifestations differ. In cyclothymia, mood fluctuation is the core symptom, whereas in KLS, the predominant feature is excessive, pathological prolonged sleep. This distinction is critical for differential diagnosis.

Treatment of KLS remains challenging due to the lack of high‐quality evidence and the disorder's unpredictable course. Although lithium and modafinil are among the most commonly used agents, their efficacy is inconsistent. The American Academy of Sleep Medicine provides a conditional recommendation for lithium use in adult KLS patients,^21^ based on very low‐quality evidence from a single open‐label study reporting reductions in episode frequency, duration, and severity, as well as improvements in quality of life and academic or occupational functioning.^22^ However, no randomized controlled trials have been conducted, and long‐term outcome data are lacking. Modafinil is used primarily as a symptomatic treatment to promote wakefulness during hypersomnolent episodes. Some case reports suggest modafinil may shorten episode duration when given early in the symptomatic phase.^7^, ^23^, ^24^ However, it has not been shown to reduce recurrence risk.

In this case, combination therapy with lithium and adjunctive modafinil yielded limited benefit. Despite treatment, the patient continues to experience recurrent hypersomnolent episodes every few months. Each episode is consistently preceded by several weeks of persistent nausea, followed by approximately 2 weeks of hypersomnolence with motivational decline, appetite loss, and weight reduction. This is followed by a recovery phase characterized by cognitive dysfunction, including impaired attention and reduced alertness, lasting several additional weeks. Together, these phases result in illness durations exceeding one month per episode, leading to significant and ongoing disruption of the patient's academic and daily functioning. These findings underscore the need for individualized treatment strategies and the development of evidence‐based pharmacologic approaches tailored to the heterogeneity and severity of KLS.

## CONCLUSION

This case represents, to our knowledge, the first diagnostically confirmed report of new‐onset KLS following COVID‐19 infection. The temporal association between infection and symptom onset suggests a possible autoimmune mechanism triggered by SARS‐CoV‐2. Given the substantial impact of KLS on daily life and social functioning, an accurate diagnosis based on clinical presentation is essential. As no consistently effective treatment has been established, further research is urgently needed to develop evidence‐based therapies tailored to the diverse manifestations of KLS.

## AUTHOR CONTRIBUTIONS

Daisuke Yoshioka treated the patient and drafted the manuscript. Takehiko Yamanashi and Masaaki Iwata critically reviewed the draft and revised it. All authors approved the final version of the manuscript.

## CONFLICT OF INTEREST STATEMENT

The authors declare no conflicts of interest.

## ETHICS APPROVAL STATEMENT

This study was conducted according to the principles of the Declaration of Helsinki.

## PATIENT CONSENT STATEMENT

Informed written consent and a signed release were obtained from the patient for the publication of this report and any accompanying images.

## CLINICAL TRIAL REGISTRATION

N/A.

## Data Availability

Data sharing is not applicable to this article as no datasets were generated or analyzed during the current study.
